# Tumor LINE-1 Methylation Level in Association with Survival of Patients with Stage II Colon Cancer

**DOI:** 10.3390/ijms18010036

**Published:** 2016-12-27

**Authors:** Marloes Swets, Anniek Zaalberg, Arnoud Boot, Tom van Wezel, Martine A. Frouws, Esther Bastiaannet, Hans Gelderblom, Cornelis J. H. van de Velde, Peter J. K. Kuppen

**Affiliations:** 1 Department of Surgery, Leiden University Medical Center, 2333 ZA Leiden, The Netherlands; m.swets@lumc.nl (M.S.); anniek.z@hotmail.com (A.Z.); m.a.frouws@lumc.nl (M.A.F.); e.bastiaannet@lumc.nl (E.B.); c.j.h.van_de_velde@lumc.nl (C.J.H.v.d.V.); 2 Department of Clinical Oncology, Leiden University Medical Center, 2333 ZA Leiden, The Netherlands; a.j.gelderblom@lumc.nl; 3 Department of Pathology, Leiden University Medical Center, 2333 ZA Leiden, The Netherlands; a.boot@lumc.nl (A.B.); t.van_wezel@lumc.nl (T.v.W.)

**Keywords:** stage II colon cancer, LINE-1, methylation, biomarker

## Abstract

Genome-wide DNA hypomethylation is associated with a worse prognosis in early-stage colorectal cancer. To measure genome-wide DNA methylation levels, long interspersed nucleotide element (LINE-1) repeats are used as a surrogate marker. Cohort studies on the clinical impact of genome-wide DNA methylation level in patients with only early-stage colon cancer, are currently lacking. This study aimed to investigate the prognostic value of LINE-1 methylation in a stage II colon cancer cohort (*n* = 164). Manual needle microdissection of tumor areas was performed on formalin-fixed paraffin-embedded tumor tissue sections followed by DNA extraction. Bisulfite converted DNA was used to assess tumor LINE-1 methylation level by qPCR. Patients with LINE-1 hypomethylated tumors had a significantly worse overall survival compared to patients with a higher level of LINE-1 tumor DNA methylation (HR 1.68, 95% CI 1.03–2.75; *p* = 0.04). This effect was more prominent in patients aged over 65 years (HR 2.00, 95% CI 1.13–3.52; *p* = 0.02), although the test for age interaction was not significant. No significant effect on recurrence-free survival was observed. Based on these results, tumor LINE-1 hypomethylation is associated with a worse overall survival in stage II colon cancer. Whether the origin of this causation is cancer-specific or age-related can be debated.

## 1. Introduction

Colorectal cancer (CRC) is one of the most commonly diagnosed malignancies across the world [1]. During the past three decades, CRC has been extensively studied regarding prognostic and predictive biomarkers, in order to establish more personalized treatment strategies. Currently, the first CRC biomarkers are entering clinical use, such as *RAS*, *BRAF*, and microsatellite mutational status. Clinically relevant biomarkers can be found at different molecular levels, and the role of epigenetics in carcinogenesis has become a focus in cancer research during the past decade. Genome-wide DNA hypomethylation is an important epigenetic alteration in cancer, including CRC. It is assumed to be an early event in the carcinogenesis and contributes to genomic instability [2,3]. The methylation status of long interspersed nucleotide element (LINE-1) repeats is used as a surrogate marker to indirectly measure global DNA methylation [4]. LINE-1 repeats are present on most of the chromosomes and make up approximately 17% of the human genome [5]. Furthermore, LINE-1 has retrotransposition activity, and upon hypomethylation LINE-1 can be reversed transcribed into DNA sequences and transpose throughout the genome. Thereby, LINE-1 can contribute to gene disruptions and genomic instability, one of the hallmarks of cancer [6,7]. Currently, tumor LINE-1 hypomethylation is intensively studied and has been observed in almost all human cancer types [8,9]. Regarding CRC, LINE-1 hypomethylation is thought to be associated with a worse prognosis, supporting its role as prognostic biomarker [10]. In addition, studies have indicated that tumor LINE-1 methylation levels correlate with tumor stage; a decrease in LINE-1 methylation, resulting in hypomethylation, was associated with more advanced disease stages [11,12]. A correlation between survival in CRC patients and tumor LINE-1 methylation status in more advanced disease stages has not been observed [12,13,14]. In the current literature, tumor LINE-1 hypomethylation in relation to clinical outcome in CRC was predominantly studied in cohorts consisting of both colon and rectal cancer. Research has provided evidence that rectal cancers differ significantly from colon tumors [15,16]. For example; rectal cancer has less microsatellite instable (MSI) tumors and fewer *BRAF* mutations when compared with colon cancer [17,18]. Many studies have supported the “two types of CRC’s” hypothesis resulting in a more definitive separation of colon and rectal cancer for scientific research and treatment strategies. For early-stage rectal cancer, the study of Benard et al. showed that LINE-1 hypomethylation was associated with a worse overall survival (OS) and higher tumor recurrence rates [14]. Large cohort studies focusing on patients with early-stage colon cancer are currently lacking. This study aimed to investigate the role of tumor LINE-1 methylation level and its relation to clinical outcome in stage II colon cancer specifically. Selecting high-risk patients with colon cancer by prognostic biomarkers is of great importance in order to avoid over-, or under treatment. Since an age-related global hypomethylation has been observed in the colon [19,20], analyses stratified by age were performed in this study. Furthermore, it is uncertain whether the proposed prognostic value of LINE-1 hypomethylation is altered by MSI [21,22]. For this reason, the *MLH1* methylation status was determined, as *MLH1* promoter methylation is the most frequently observed cause of MSI in sporadic colon tumors [23].

## 2. Results

### 2.1. Patient Characteristics

In total, 181 patients were included based on the availability of FFPE tissue blocks. After manual needle microdissection, a sufficient amount of tumor tissue was collected and successful DNA extraction was performed for 164 out of 181 patients. Table 1 summarizes the clinical, pathological and treatment characteristics of the patients included for analyses (*n* = 164).

### 2.2. Methylation Assay Verification

To ensure reproducibility and a good performance of the LINE-1 methylation assay, several quality checks were performed. The inter assay variation of the control samples, showed minimal inter-plate variations: (<7%). A standard curve, using proportions of the universally methylated and unmethylated DNA was constructed and showed to be highly reproducible, with *r^2^* ≥ 0.96 (Figure S1b). To control variation, each DNA sample was run in triplicate. As indicated in Figure S1c, the three angles (α) of each patient sample were highly comparable.

### 2.3. LINE-1 Methylation Level and Patient Survival

**Figure 1:**
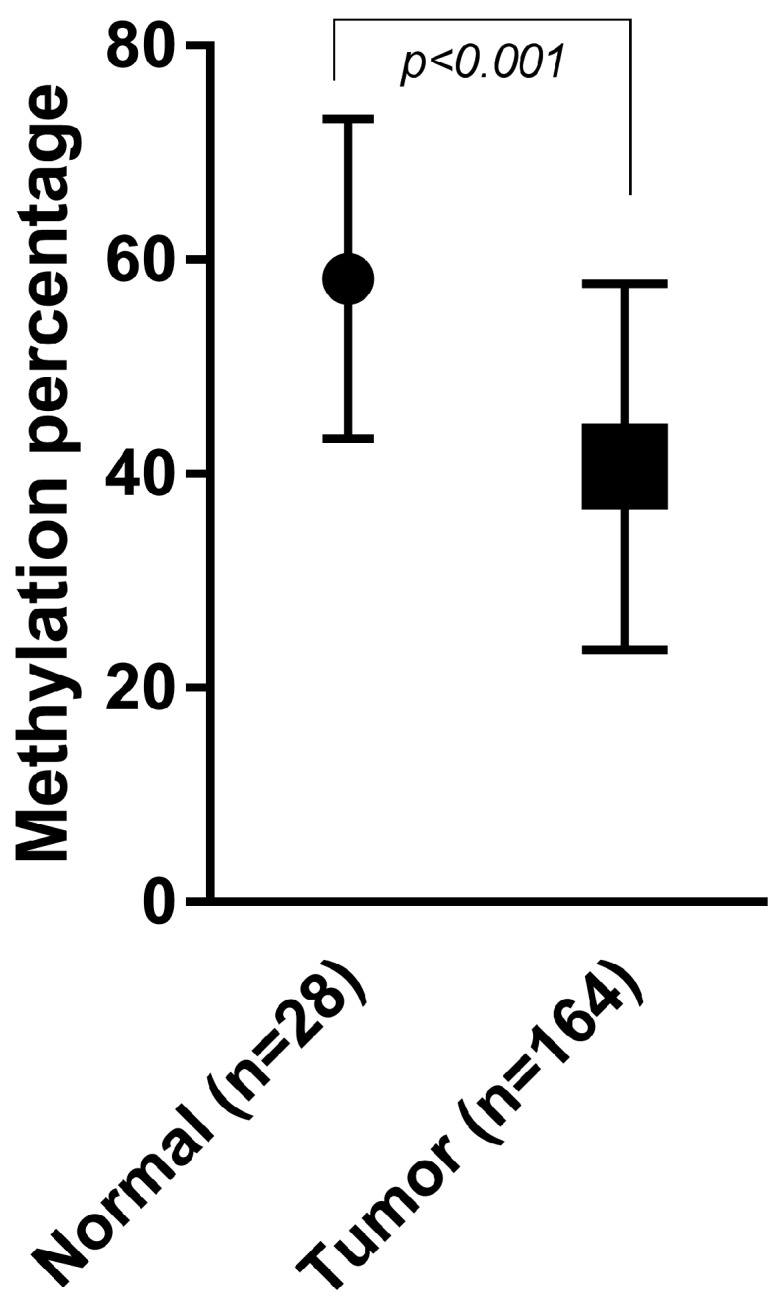
Average LINE-1 methylation level in normal and tumor epithelium. Mean methylation level of 28 normal colon epithelial tissues (58.2% ± SD 14.9%) compared with 164 stage II colon tumor tissue samples (40.7% ± SD 17.7%). Error bars represent the standard deviation of the mean.

As shown in Figure 1, normal colon tissues (*n* = 28) showed significantly different relative LINE-1 methylation levels (58.2% ± SD 14.9%) compared with tumor tissue of the total patient cohort of 164 patients (40.7% ± SD 17.1%) with *p* < 0.001.

Based on the calculated methylation levels of tumor DNA, a methylated and a hypomethylated group were defined for comparison. The tumor LINE-1 hypomethylated group consisted of patients with the lowest one third of the calculated methylation levels (median relative LINE-1 methylation level; 24.1%). The tumor LINE-1 methylated group consisted of patients with the upper two third of the methylation levels (median relative LINE-1 methylation level; 45.3%). The mentioned percentages are not reflecting the absolute percentages, but a relative measure in comparison to the used standards (Figure S1).

**Figure 2:**
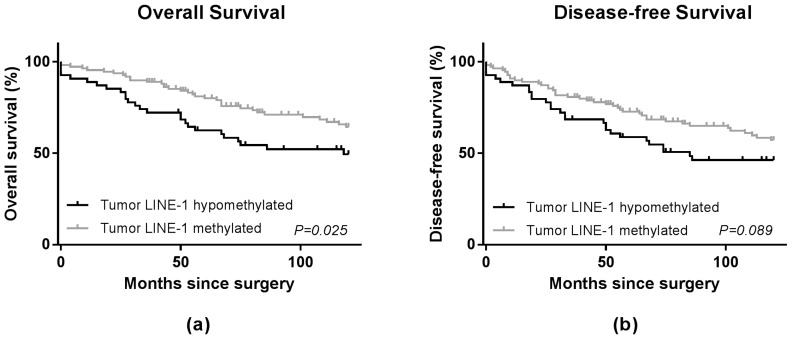
Survival curves for overall survival (**a**) and disease-free survival (**b**) in 164 patients with stage II colon cancer. Solid black line represents patients with tumor LINE-1 hypomethylation. The grey line represents patients without tumor LINE-1 hypomethylation. The *p*-value in the graphs represents the Log-Rank value.

No significant differences were observed between the hypomethylated LINE-1 group (*n* = 54) and methylated LINE-1 group (*n* = 110), regarding the clinical and pathological characteristics (Table 1). As shown in the Kaplan-Meier survival curve (Figure 2), patients with hypomethylated LINE-1 tumors had a significantly worse OS compared with patients in the methylated LINE-1 group with a significant Log-Rank test. The Cox regression model for overall survival showed a hazard ratio (HR) of 1.75 (95% CI 1.09–2.81; *p* = 0.02, Table 2). Multivariate analysis, adjusted for age, gender and *MLH1* methylation status of the OS showed a HR of 1.68 (95% CI 1.03–2.75; *p* = 0.04, Table 2). No statistically significant difference for disease-free survival (DFS) was observed in the Kaplan-Meier survival curve (Figure 2), univariate (HR 1.52, 95% CI 0.97–2.40; *p* = 0.07) or multivariate analysis (HR 1.43, 95% CI 0.90–2.29; *p* = 0.13, Table 2). Furthermore, no significant difference between patients in the hypomethylated and methylated group was found for relapse-free survival (RFS) in univariate and multivariate analyses (Table 2).

**Figure 3:**
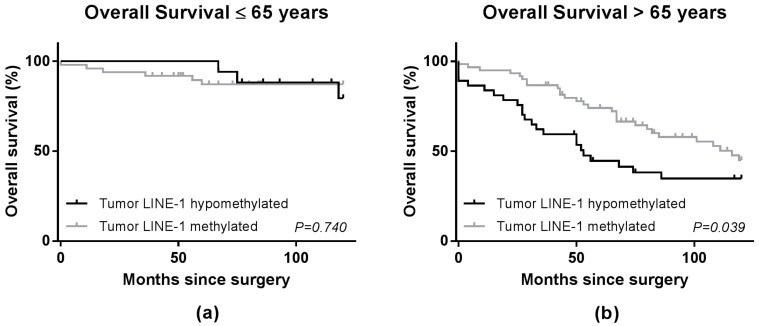
Survival curves for overall survival in colon cancer stage II patients stratified by age in two groups; ≤65 years at operations (*n* = 66) (**a**) and >65 years at operation (*n* = 98) (**b**). Solid black line represents patients with tumor LINE-1 hypomethylation. The grey line represents patients without tumor LINE-1 hypomethylation. The *p*-value in the graphs represents the Log-Rank value.

Analyses for tumor LINE-1 hypomethylation stratified by age, ≤65 years at operation and >65 years at operation, were performed since global hypomethylation was reported to be associated with increasing age [19]. As shown in Figure 3 and Table 3, a significantly worse OS for patients with hypomethylated LINE-1 tumors was observed in the patients older than 65 years at operation in the univariate (HR 1.93, 95% CI 1.15–3.26; *p* = 0.01) and multivariate analysis (HR 2.00, 95% CI 1.13–3.52; *p* = 0.02). Furthermore, in multivariate analysis of DFS, a significant unfavorable prognosis was found for patients older than 65 with LINE-1 hypomethylated tumors (HR 1.76, 95% CI 1.02–3.05; *p* = 0.04). No significant differences were found in RFS (Table 3). In contrast, in patients with colon cancer who were younger than 65 years of age at time of diagnosis, no significant differences in OS, DFS or RFS were observed regarding LINE-1 methylation levels (Table 3). The Wald tests showed non-significant p-values for interaction (Table 3), which suggests that the difference found between the two age groups could be based on chance.

*MLH1* promoter hypermethylation was found in 18.3% of the patients. *MLH1* promoter methylation is the most commonly observed alteration causing MSI in sporadic colon tumors [23], and consequently the tumors with *MLH1* promoter methylation are likely to represent most of the MSI tumors of the cohort, although we did not evaluate MSI independently. The effect of tumor LINE-1 methylation was analyzed in patients with and without *MLH1* methylated promoters separately. Corresponding with the total study cohort, an unfavorable clinical outcome was observed in patients with LINE-1 hypomethylated tumors (HR 1.85, 95% CI 1.10–3.11; *p* = 0.02) within the patients without *MLH1* promoter hypermethylation (Table 2). Multivariate analysis of the OS in this group showed a HR of 1.56 (95% CI 0.93–2.63; *p* = 0.09, Table 2). LINE-1 methylation did not correlate with survival in patients with *MLH1* promoter hypermethylation. Due to the low number of patients with methylated *MLH1* promoters (*n* = 30), no multivariate analysis was performed (Table 2).

## 3. Discussion

Genome-wide DNA hypomethylation is an important epigenetic alteration in CRC [2,3]. Furthermore, it has been proposed that loss of global DNA methylation is strongly associated with hypomethylated LINE-1 elements, subsequently LINE-1 methylation serves as a surrogate marker for overall DNA methylation status [4]. Accurate identification and isolation of tumor cells is highly important in studying tumor LINE-1 methylation. Considering tumor-associated stromal cells could influence the measured tumor methylation levels and consequently bias the results. A study conducted by Irahara et al. demonstrated that results regarding LINE-1 methylation levels obtained with manual needle microdissection were comparable to isolating tumor cells by laser capture technique [24]. Accordingly, needle microdissection of tumor cells was applied in this study; consequently, a minimal contaminating effect of DNA from stromal cells could be expected.

Many studies have reported on the effect of LINE-1 hypomethylation, showing that tumor LINE-1 hypomethylation results in a worse prognosis in CRC patients, especially in patients with proximal located colon tumors [10,11,12,14,25]. Interestingly, the majority of the performed studies did not reveal a relation between LINE-1 hypomethylation and clinical outcome in more advanced disease stages [12,13,14]. For that reason, only patients with stage II colon cancer were included in this study to evaluate the assumed prognostic value of LINE-1 hypomethylation in early-stage colon cancer without the interference of patients with advanced disease stages or patients with rectal cancers. In line with the above-mentioned studies, we observed a significantly worse overall survival in patients with stage II colon tumors with LINE-1 hypomethylation, compared to those with tumors with higher LINE-1 methylation levels, supporting the role of LINE-1 as a prognostic biomarker. In contrast, no significant differences were observed in DFS and RFS among patients with hypomethylated and methylated tumors. Based on the results obtained in this study, a specific role for LINE-1 hypomethylation as a biomarker for colon cancer disease progression could not be suggested. Combined with the fact that no correlation between LINE-1 hypomethylation and survival was observed in more advanced disease stages, global loss of DNA methylation appears more as an early event in colon cancer formation rather than during disease progression [26,27,28]. The contribution of LINE-1 hypomethylation in colon cancer formation is supported by the study of Pavicic et al., in which LINE-1 methylation levels were evaluated in normal epithelial tissues of patients with hereditary nonpolyposis colorectal cancer (HNPCC), familiar colorectal cancer and sporadic cancer [29]. They found the lowest LINE-1 methylation levels in normal mucosa of patients with familial CRC, suggesting that lower levels of LINE-1 methylation predispose normal tissue to cancer development. Furthermore, patients diagnosed with serrated polyps (at risk of synchronous CRC development) did show LINE-1 hypomethylation in the normal adjacent colon mucosa [30]. Moreover, studies revealed that colon mucosa shows an age-related global hypomethylation [19,28]. Models have been developed to predict chronological age from DNA methylation [31]. A number of studies indicate that a discrepancy between chronological age and age predicted based on DNA methylation patterns (i.e., predicted age based on methylation exceeding chronological age) has been associated with an increased mortality risk [32,33]. This could explain the observed worse OS, without differences in DFS and RFS, in patients with LINE-1 hypomethylated tumors, especially in patients aged over 65 years*.* Accordingly, LINE-1 hypomethylation might be associated with an increased mortality risk in general, rather than a more aggressive tumor. This suggests that LINE-1 hypomethylation does not contribute to disease progression, although a prognostic role for LINE-1 hypomethylation in a more general way could be considered. Notably, no statistical interaction between the age groups was observed; the effect of age on the association of LINE-1 methylation status and survival could be based on chance. Therefore, conclusions on LINE-1 hypomethylation in combination with age have to be drawn carefully.

Conflicting results have been published regarding tumor LINE-1 hypomethylation in combination with MSI status in CRC in relation to survival [21,22]. *MLH1* promoter methylation is the most commonly observed alteration causing MSI in sporadic colon tumors [23]. In the general population, 15% of the sporadic colon tumors are MSI [20,34,35,36], consistent with the observed 18.5% of the tumors in our cohort. Unfortunately, the number of patients in this subgroup was too small to draw a conclusion on the effect of LINE-1 hypomethylation in patients with MSI colon tumors. Larger study cohorts will be needed to firmly analyze MSI in combination with LINE-1 methylation levels.

Tumor LINE-1 hypomethylation, was used as surrogate marker for global DNA hypomethylation in this study. Based on the results of this study, a role for global DNA hypomethylation in colon cancer development, rather than LINE-1 methylation level as a biomarker for disease progression, could be suggested. Additional studies to further evaluate whether or not LINE-1 hypomethylation has a specific role in disease progression in stage II colon cancer will be needed. Large cohorts will be essential, considering the low tumor recurrence rates in patients with early-stage colon cancer. Besides a surrogate marker for genome-wide hypomethylation, LINE-1 hypomethylation could result in increased retrotransposition activity and integration of LINE-1 elements near oncogenes or tumor suppressor genes and may influence cancer development or disease progression [37,38]. Further research will be essential to fully unravel these complex mechanisms in the scope of colon cancer development and disease progression.

## 4. Materials and Methods

### 4.1. Patient Selection

Formalin-fixed paraffin-embedded (FFPE) tumor tissues were collected from patients with stage II colon cancer who underwent radical resection of the primary tumor between 1991 and 2011 at the Leiden University Medical Center. Patients with a history of cancer other than basal cell carcinoma, patients that received radiotherapy and/or chemotherapy prior to resection, patients with multiple synchronous colon tumors and patients with rectal cancer were excluded. Based on availability of paraffin tissue blocks, 181 patients were included. Clinical, pathological and follow-up data were collected in a retrospective manner from hospital records. Patient information was anonymized and de-identified prior to analysis. This research was performed according to the national ethical guidelines (“Code for Proper Secondary Use of Human Tissue”, Federation of Medical Scientific Societies). For the comparison of LINE-1 methylation levels in tumor and adjacent normal epithelium, for 28 patients, normal tissue was also collected.

### 4.2. DNA Extraction from Formalin-Fixed Paraffin-Embedded Tumor Tissues and Bisulfite Conversion

In order to reduce the amount of tumor stromal tissue components, tumor areas (>80% neoplastic epithelial cells) on haematoxylin and eosin-stained tumor sections were marked. Of each patient block, five FFPE tumor tissue sections of 7 µm were deparaffinized and stained with haematoxylin, followed by manual needle microdissection of the marked areas. After microdissection, genomic DNA was extracted from the collected tumor material using the Microlab starLET IVD robot (Hamilton Robotics, Bonaduz, Switzerland) and quantified using a Qubit^®^ 2.0 Fluorometer according to the Qubit ds DNA HS Assay kit protocol (Invitrogen™, Eugene, OR, USA). Bisulfite conversion was performed with EZ DNA Methylation Gold kit (Zymo Research Corp, Orange, CA, USA) on the isolated genomic DNA, according to the manufacturer’s instructions, using an input of 50 ng of DNA. Bisulfite converted DNA was eluted in 15 µL Milli-Q purified water.

### 4.3. Quantitative Real-Time PCR

The PCR primers used in this study amplified the target sequence independent from methylation status. In addition, minor-groove-binding (MGB) methylation specific probes were used as previously used by Sunami et al. [12]. Primer sequences were as follow; 5′-GGGTTTATTTTATTAGGGAGTGTTAGA-3′ (forward), 5′-TCACCCCTTTCTTTAACTCAAA-3′ (reverse). The probes were labelled with Hexachloro-Fluorescein (HEX) and 6-fluorescein amidite (FAM) sequences were as follows; Allele 1-FAM-5′-TGCGCGAGTCGAAGT-3′-MGB-BHQ (methylated-specific) and Allele 2-HEX-5′-TGTGTGAGTTGAAGTAGGG-3′-MGB-BHQ (unmethylated-specific) (Biolegio, Nijmegen, The Netherlands). Real-time PCR was performed in a final reaction volume of 10 µL consisting of 1 µL bisulfite converted DNA template, 200 µM deoxynucleotide triphosphates (dNTPs), 1.9 µM MgCl_2_, 1× PCR Gold Buffer, 1 unit of AmpliTaq Gold DNA Polymerase^®^ (Applied Biosystems, Foster City, CA, USA), 0.4 μM of the forward and reverse primer, 0.25 µM of MGB probes. The following protocol was executed: 5 min 95 °C, 7× (15 s 95 °C, 30 s 64 °C with a 1 °C decrement every cycle, 20 s 72 °C); 39× (15 s 95 °C, 30 s 52 °C, 20 s 72 °C); 1 min 60 °C and a melt curve from 65 to 95 °C with a increment of 0.5 °C for 10 s. Quantitative PCR reactions were run on a CFX96™ Real-Time system C1000™ Thermal cycler (BioRad, Benicia, CA, USA). All reactions were performed in triplicate. Controls used for LINE-1 methylation assays were universally methylated DNA (Millipore, Billerica, MA, USA) and universally unmethylated DNA obtained by repeated whole genome amplification of peripheral blood lymphocyte DNA (Repli-g kit Qiagen, Valencia, CA, USA). A standard curve was generated by the use of a mix with proportions (0%, 10%, 25%, 40%, 60%, 75%, 90% and 100%) of the universally methylated and unmethylated DNA and was used for quantification of LINE-1 methylation levels in the patient samples.

### 4.4. Analysis of LINE-1 Methylation Levels

Relative fluorescence units (RFU) were used to calculate the level of methylated DNA. Samples with higher quantities of amplified methylated or unmethylated DNA have higher RFU values for Allele 1-FAM or Allele 2-HEX respectively. A standard curve was generated with the RFU of the Allele 1-FAM-probe and Allele 2-HEX-probe which were obtained by using a mixture of universally methylated and unmethylated DNA in different ratios as indicated in the previous paragraph. The RFU for both probes were plotted followed by the calculation of the angle (α) (Figure S1a), as a measure of the ratio between methylated-unmethylated LINE-1 elements. For each sample the mean α of the triplicates was calculated. By using the standard curve, the LINE-1 methylation level was determined by looking up the corresponding angle (α).

### 4.5. MLH1 Methylation Status

MLH1 methylation analyses were performed using the bisulfite converted tumor DNA (described above) according to the protocols developed at the molecular diagnostics of the department of Pathology at the LUMC as described by van Roon et al. [39].

### 4.6. Statistical Analysis

Statistical analyses were performed using the statistical package SPSS (version 20.0 for Windows; SPSS Inc.). Student’s T test and the Chi-squared test were used for the evaluation of the association between LINE-1 methylation levels and clinical-pathological parameters. Overall survival (OS) was defined as time of surgery until death. Disease-free survival (DFS) was defined as time of surgery until relapse of the disease or death, whichever came first. The definition of recurrence-free survival (RFS) was time of surgery until local or distant recurrences, whichever came first. Deaths were censored in this analysis. For survival probabilities the Kaplan-Meier method was used, for comparison of the survival curves the Log-Rank test was used. Kaplan-Meier curves were censored at 10 years of follow-up. Univariate and multivariate Cox regression analyses were performed to evaluate the differences in OS, DFS and RFS in patients with methylated versus patients with hypomethylated LINE-1 elements. The tumor LINE-1 hypomethylated group consisted of patients with the lowest one third of the calculated methylation levels. The tumor LINE-1 methylated group consisted of patients with the upper two third of the methylation levels. To investigate differential association of LINE-1 methylation level in survival by molecular subtype and age, pre-specified stratified analyses were performed for *MLH1* methylation status and age followed by interaction analysis which was assessed with the Wald test. Covariates entered in the multivariate model were age, sex, and *MLH1* methylation status. For all tests, a *p*-value of <0.05 was considered as statistically significant.

## Figures and Tables

**Table 1:** Baseline clinical and pathological characteristics for the total cohort (*n* = 164) and stratified for tumor long interspersed nucleotide element (LINE-1) methylation status. Data are presented as *n* (%) or as median ± SD.

	Total *n* = 164 (%)	LINE-1		*p*-Value
		Hypomethylated ^a^ *n* = 54 (%)	Methylated ^b^ *n* = 110 (%)	
**Gender**				
Male	86 (52.4)	30 (55.6)	56 (65.1)	0.58
Female	78 (47.6)	24 (44.4)	54 (49.1)	
**Age median**	68.0 (±13.6)	69.5 (±12.0)	68.0 (±14.3)	0.16
**Age Groups**				
≤65	66 (40.2)	17 (31.5)	49 (44.5)	0.11
>65	98 (59.8)	37 (68.5)	61 (55.5)	
**Grade**				
Good	32 (19.5)	11 (20.4)	21 (19.1)	0.24
Moderate	79 (48.2)	30 (55.6)	49 (48.2)	
Poor	20 (12.2)	7 (13.0)	13 (11.8)	
Unknown	33 (9.8)	6 (11.1)	27 (24.5)	
**Location**				
Right	74 (45.1)	22 (40.7)	52 (47.3)	0.43
Left	90 (54.9)	32 (59.3)	58 (52.7)	
***MLH1* Promoter**				
Methylated	30 (18.3)	6 (11.1)	24 (21.8)	0.10
Unmethylated	134 (81.7)	48 (88.9)	86 (78.2)	
**Adjuvant Therapy**				
No	156 (95.1)	50 (92.6)	106 (96.4)	0.29
Yes	8 (49)	4 (7.4)	4 (3.6)	

Data are presented as median ± SD or *n* (%); ^a^: Hypomethylated group includes 1/3 of the patient cohort with the lowest methylation levels; ^b^: Methylated group includes 2/3 of the patient cohort with higher methylation levels.

**Table 2:** Univariate and multivariate analyses for overall survival, disease-free survival and recurrence-free survival comparing tumor LINE-1 methylated and tumor LINE-1 hypomethylated, in stage II colon cancer in the total cohort and stratified by *MLH1* status.

	Patients *n* = 164	Univariate		Multivariate ^a^	
		HR (95% CI)	*p*-Value	HR (95% CI)	*p*-Value
**Overall Survival**					
LINE-1 Methylated	110	1.00 (*reference*)	0.02	1.00 (*reference*)	0.04
LINE-1 Hypomethylated	54	1.75 (1.09–2.81)		1.68 (1.03–2.75)	
**Disease-free Survival ^b^**					
LINE-1 Methylated	110	1.00 (*reference*)	0.07	1.00 (*reference*)	0.13
LINE-1 Hypomethylated	54	1.52 (0.97–2.40)		1.43 (0.90–2.29)	
**Recurrence-free Survival ^c^**					
LINE-1 Methylated	110	1.00 (*reference*)	0.37	1.00 (*reference*)	0.33
LINE-1 Hypomethylated	54	1.40 (0.66–2.97)		1.46 (0.68–3.15)	
**Overall Survival by *MLH1* Status**					
***MLH1* Unmethylated**	134				
LINE-1 Methylated	86	1.00 (*reference*)	0.02	1.00 (*reference*)	0.09
LINE-1 Hypomethylated	48	1.85 (1.10–3.11)		1.56 (0.93–2.63)	
***MLH1* Methylated**	30				
LINE-1 Methylated	24	1.00 (*reference*)	0.89	-	-
LINE-1 Hypomethylated	6	1.09 (0.29–4.07)		-	

^a^: Adjusted for age, gender, *MLH1* methylation status; ^b^: Disease-free survival defined as time from operation until local-regional recurrence, distant recurrence or death whichever came first; ^c^: Recurrence- free period define as time from operation until any recurrence (local-regional recurrence and/or distant recurrence) whichever came first.

**Table 3:** Hazard Ratio for overall survival, disease-free survival and recurrence-free survival comparing methylated LINE-1 and hypomethylated LINE-1 stage II colon tumors stratified by age. Hazard ratios are displayed for both Cox proportional hazard univariate and multivariate analysis with 95% confidence interval. The Wald test was performed to calculated the *p*-value for interaction.

	Patients *n* = 164	Univariate		Multivariate ^a^		*p*-Value for Interaction ^b^
		HR (95%CI)	*p*-Value	HR (95% CI)	*p*-Value	
**Overall Survival**						
≤65 years	66					0.24
LINE-1 Methylated	49	1.00 (*reference*)	0.82	1.00 (*reference*)	0.80	
LINE-1 Hypomethylated	17	0.81 (0.22–3.04)		0.84 (0.23–3.16)		
>65 years	98					
LINE-1 Methylated	61	1.00 (*reference*)	0.01	1.00 (*reference*)	0.02	
LINE-1 Hypomethylated	37	1.93 (1.15–3.26)		2.00 (1.13–3.52)		
**Disease-Free Survival ^c^**						
≤65 years	66					0.31
LINE-1 Methylated	49	1.00 (*reference*)	0.76	1.00 (*reference*)	0.71	
LINE-1 Hypomethylated	17	0.84 (0.27–2.56)		0.71 (0.26–2.53)		
>65 years	98					
LINE-1 Methylated	61	1.00 (*reference*)	0.05	1.00 (*reference*)	0.04	
LINE-1 Hypomethylated	37	1.67 (1.00–2.77)		1.76 (1.02–3.05)		
**Recurrence-Free Survival**						
≤65 years	66					0.35
LINE-1 Methylated	49	1.00 (*reference*)	0.93	1.00 (*reference*)	0.99	
LINE-1 Hypomethylated	17	0.94 (0.25–3.55)		1.00 (0.26–3.08)		
>65 years	98					
LINE-1 Methylated	61	1.00 (*reference*)	0.28	1.00 (*reference*)	0.11	
LINE-1 Hypomethylated	37	1.67 (0.66–4.23)		2.32 (0.83–6.52)		

^a^: Adjusted for gender, *MLH1* methylation status; ^b^: *p*-Value for interaction was calculated for univariate analysis; ^c^: Disease-free survival defined as time from operation until local-regional recurrence, distant recurrence or death, whichever came first ^d^: Recurrence-free period define as time from operation until any recurrence (local-regional recurrence and/or distant recurrence), whichever came first.

## Supplementary Materials

Supplementary materials can be found at www.mdpi.com/1422-0067/18/1/36/s1.

## Abbreviations

CRC	Colorectal cancer
DFS	Disease-free survival
HEX	Hexachloro-Fluorescein
FAM	6-Fluorescein amidite
FFPE	Formalin-fixed paraffin-embedded
LINE-1	Long interspersed nucleotide element
MGB	Minor-groove-binding
MSI	Microsatellite instable
OS	Overall survival
RFS	Recurrence-free survival
RFU	Relative fluorescence units
(q)PCR	(Quantitative) polymerase chain reaction
