# Supplementary material for: Tumor LINE-1 Methylation Level in Association with Survival of Patients with Stage II Colon Cancer
Source: Int J Mol Sci. 2016 Dec 27;18(1):36. doi: 10.3390/ijms18010036 (PMC5297671; doi:10.3390/ijms18010036)
Supplement: Supplementary file 1 [file ijms-18-00036-s001.pdf]

Supplementary Materials: Tumor LINE-1 Methylation Level in Association with Survival of Patients with Stage II Colon Cancer

Marloes Swets, Anniek Zaalberg, Arnoud Boot, Tom Van Wezel, Martine A. Frouws, Esther Bastiaannet, Hans Gelderblom, Cornelis J. H. Van De Velde and Peter J. K. Kuppen

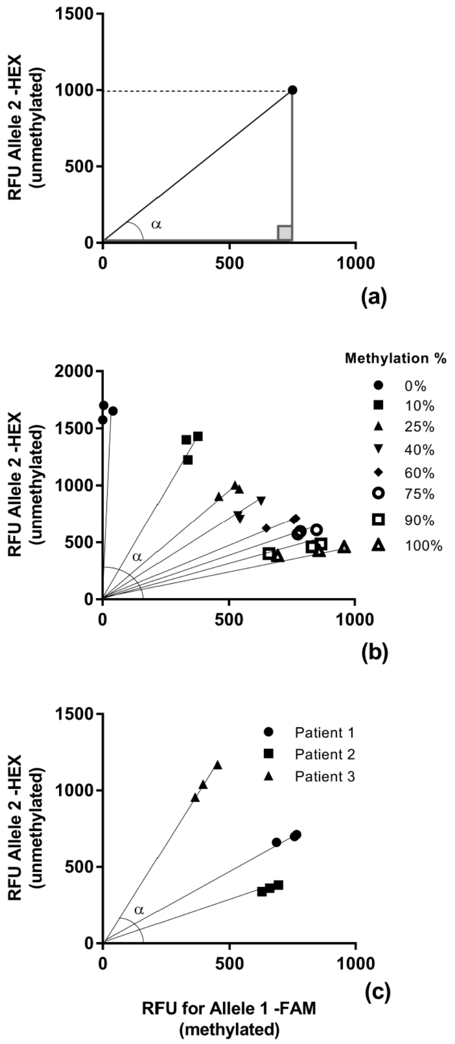

Figure S1. Explanation of the calculation method for determining LINE-1 methylation level. The x-axis shows the intensity of the qPCR signal for the methylation specific probe (Allele 1-FAM) expressed in the relative fluorescence units (RFU). The y-axis shows the intensity of the qPCR signal for the unmethylated specific probe (Allele 2-HEX) expressed in RFU; (**a**) The Pythagorean theorem was used to calculate angle α (α = inverse tangent x (RFU Allele 2-HEX/RFU Allele 1-FAM)); (**b**) A standard curve was generated by the use of a mixture of universally methylated and unmethylated DNA. Triplicates of the samples were used. The graph shows the allelic discrimination. The percentages represent the percentage of methylated DNA. As indicated in this graph the higher the α the lower the methylation level; (**c**) Representative patient samples. Based on the calculated angle α the level of LINE-1 methylation could be determined.
